# Identification and characterization of novel conserved RNA structures in Drosophila

**DOI:** 10.1186/s12864-018-5234-4

**Published:** 2018-12-11

**Authors:** Rebecca Kirsch, Stefan E. Seemann, Walter L. Ruzzo, Stephen M. Cohen, Peter F. Stadler, Jan Gorodkin

**Affiliations:** 1 Center for non-coding RNA in Technology and Health, University of Copenhagen, Grønnegårdsvej 3, Frederiksberg C, DK-1870 Denmark; 2 Department of Veterinary and Animal Science, University of Copenhagen, Grønnegårdsvej 3, Frederiksberg C, DK-1870 Denmark; 3 School of Computer Science and Engineering, University of Washington, Box 352350, Seattle, 98195-2350 WA USA; 4 Department of Genome Sciences, University of Washington, Box 355065, Seattle, 98195-5065 WA USA; 5 Fred Hutchinson Cancer Research Center, 1100 Fairview Ave. N., Seattle, 98109-1024 WA USA; 6 Department of Cellular and Molecular Medicine, University of Copenhagen, Blegdamsvej 3, Copenhagen N, DK-2200 Denmark; 7 Bioinformatics Group, Department of Computer Science, and Interdisciplinary Center for Bioinformatics, Universität Leipzig, Härtelstraße 16–18, Leipzig, D-04107 Germany; 8 Max Planck Institute for Mathematics in the Sciences, Inselstraße 22, Leipzig, D-04103 Germany; 9 Faculdad de Ciencias, Universidad Nacional de Colombia, Sede Bogotá, Ciudad Universitaria, Bogotá, COL-111321 D.C. Colombia; 10 Department of Theoretical Chemistry, University of Vienna, Währinger Straße 17, Vienna, A-1090 Austria; 11 Santa Fe Institute, 1399 Hyde Park Rd., Santa Fe, NM87501 USA

**Keywords:** Non-coding RNA, RNA secondary structure prediction, *Drosophila melanogaster*, CMfinder, Gene expression, Development

## Abstract

**Background:**

Comparative genomics approaches have facilitated the discovery of many novel non-coding and structured RNAs (ncRNAs). The increasing availability of related genomes now makes it possible to systematically search for compensatory base changes – and thus for conserved secondary structures – even in genomic regions that are poorly alignable in the primary sequence. The wealth of available transcriptome data can add valuable insight into expression and possible function for new ncRNA candidates. Earlier work identifying ncRNAs in *Drosophila melanogaster* made use of sequence-based alignments and employed a sliding window approach, inevitably biasing identification toward RNAs encoded in the more conserved parts of the genome.

**Results:**

To search for conserved RNA structures (CRSs) that may not be highly conserved in sequence and to assess the expression of CRSs, we conducted a genome-wide structural alignment screen of 27 insect genomes including *D. melanogaster* and integrated this with an extensive set of tiling array data. The structural alignment screen revealed ∼30,000 novel candidate CRSs at an estimated false discovery rate of less than 10%. With more than one quarter of all individual CRS motifs showing sequence identities below 60%, the predicted CRSs largely complement the findings of sliding window approaches applied previously. While a sixth of the CRSs were ubiquitously expressed, we found that most were expressed in specific developmental stages or cell lines. Notably, most statistically significant enrichment of CRSs were observed in pupae, mainly in exons of untranslated regions, promotors, enhancers, and long ncRNAs. Interestingly, cell lines were found to express a different set of CRSs than were found in vivo. Only a small fraction of intergenic CRSs were co-expressed with the adjacent protein coding genes, which suggests that most intergenic CRSs are independent genetic units.

**Conclusions:**

This study provides a more comprehensive view of the ncRNA transcriptome in fly as well as evidence for differential expression of CRSs during development and in cell lines.

**Electronic supplementary material:**

The online version of this article (10.1186/s12864-018-5234-4) contains supplementary material, which is available to authorized users.

## Background

Over the last decade our understanding of the functioning of eukaryotic genomes has changed profoundly. The vast majority of the DNA sequence is transcribed into RNA, and protein-coding sequences comprise only a fraction of the informational content encoded by RNA [1, 2]. This is true for mammals as well as for simple model organisms such as yeast [3].

The functions of the vast majority of these transcripts are unknown. The fact that much of the transcriptional output is poorly conserved at the sequence level initially led to doubts that this pervasive transcription was more than just irrelevant “Junk RNA” [4]. A growing body of evidence, however, showed that many non-coding transcripts are under selection acting at the RNA level. One line of evidence is based on the conservation of gene structures [5]. Another traces the evolution of RNA secondary structure elements [6].

Many ncRNAs compiled in the Rfam database exhibit well-conserved RNA secondary structures. Independent ncRNAs such as transfer RNAs (tRNAs), small nuclear RNAs (snRNAs), ribosomal RNAs (rRNAs), small nucleolar RNA (snoRNAs), and microRNAs (miRNAs) constitute only a minute fraction of the genome. However, structured RNA motifs are much more widespread. Regulatory features of mRNAs, such as internal ribosome entry site (IRES) and selenocysteine insertion sequence (SECIS) elements, aptamer domains of riboswitches, or the autoregulatory domains of many messenger RNAs (mRNAs) that encode ribosomal proteins also have recognizable secondary structures [7].

The presence of a stable secondary structure is not in itself a sufficient indication that the RNA has a function: Random RNA sequences typically fold into highly complex secondary structures that are not statistically different from known functional elements [8–11]. Therefore, it is necessary to assess the evolutionary conservation of secondary structures.

A variety of computational methods have been developed to identify negative selection acting on RNA structure. Methods starting from multiple sequence alignments include qrna [12], AlifoldZ [13], EvoFold [14], RNAz [15], and SISSIz [16]. Their main limitation is the need for reliable sequence-based multiple sequence alignments. This can be partially overcome by methods that align or re-align (presumably) homologous sequences using sequence and structure simultaneously. A widely used tool of this type is CMfinder [17, 18]. A pipeline centered around FoldAlign [19] uses the same basic logic. We refer to [20] for a review. REAPR is an improved method that shares the idea of structure-based re-alignment of regions with the approach pursued here. It achieved a doubling of sensitivity and confirmed a substantial number of its predictions as transcripts [21].

Studies on ncRNA gene families of fruit flies have a long history. Well understood and well conserved ncRNA families, such as miRNAs [22], have frequently been used as model systems to study ncRNA evolution [23, 24]. Several previous experimental as well as computational surveys have suggested that the fruit fly and related insect species still harbor large numbers of unexplored ncRNAs. For example, thousands of long ncRNAs (lncRNAs) were found using deep sequencing [25–27]. A study focusing on 3’-untranslated regions (UTRs) found 184 ncRNA clusters [28]. A quarter of the genomic regions that currently lack annotated genes, i.e., that are currently considered “intergenic”, show transcriptional activity (according to the intersection of the current annotation [29] and a genome-wide tiling array study [30]). Most likely, these regions harbor still undescribed transcripts. We consider this value of one quarter as a lower bound since it is unlikely that any individual study captures the complete transcriptome.

A computational screen for structured RNAs using RNAz identified about 16,000 candidate RNA elements with an estimated false discovery rate (FDR) of about 40% [31]. However, RNAz evaluates only the most conserved parts of genomic sequence alignments and is optimized for specificity. Subsequent computational analyses of mammalian genomes indicate that the number of functional RNAs is most likely considerably higher: Up to a fifth of the genome may be under selection for RNA structure, but only a tenth of these loci show evidence of selection for conservation of nucleic acid sequence [6, 18, 32].

Several computational surveys of structured RNAs [15] have confirmed the presence of large numbers of conserved structured RNA elements in fruit flies, notably a more detailed RNAz-based screen [31] and a comparison of several grammar-based methods [33]. RNAz likely underestimates the number of conserved RNA structures in flies similar to the situation in mammals. A subsequent study concentrating on coding regions, furthermore, suggests that these also harbor many superimposed RNA structures [34]. CMfinder takes this approach a step further by joint folding and structure-based re-alignment of genome sequences [35]. To date, the newest generation of computational ncRNA screening methods have not been applied to fly genomes. We close this gap here and provide a map of conserved RNA structures (CRSs) in the fruit fly *Drosophila melanogaster*. Furthermore, we associate CRSs with expression across all developmental stages in fly as well as expression in cell lines, which has not been done before.

## Results

### Summary of the CMfinder screen

We predicted CRSs on the genomic sequences of 23 drosophilid and four additional insect species extracted from the UCSC Genome Browser (see “Methods” section for details). Multiple alignment blocks shorter than 50 bp or containing fewer than three sequences were removed. Within each alignment block, the sequence-based alignment was ignored and the unaligned sequences were fed to CMfinder. A total of 345,285 CMfinder predictions passed our filter criteria including a minimum *pscore* [35] *p*>50 and a minimum element size of 30 nt (see “Methods” section for details). Salient features of these candidates are summarized in Additional file 1: Figure S1: The majority of the predictions had folding energies in the range of about −10 kcal/mol and were shorter than 100 nt.

This fits well with the properties of most of the small structured ncRNA genes and most of the well-known functional RNA elements in mRNAs. Their GC content lay mainly between 30 and 60% with a tail more pronounced towards 20%, commensurate with the comparatively low overall GC content in drosophilid genomes [36]. A sequence identity of 40 to 80% reflects the lower sequence conservation in structurally conserved RNAs. Most predictions were found in 17 to 23 of the 27 genomes examined. All CRSs are available at https://rth.dk/resources/rnannotator/crs/insect/.

Out of the 345,285 initial predictions, 12,421 overlapped an annotated repeat by at least 50% of their length. These were removed from further processing because the input alignments are unreliable in repetitive regions (see e.g. [37, 38]). While the initial predictions were obtained with a uniform cut-off for CMfinder’s *pscore*, previous applications of CMfinder to vertebrate genomes have shown that the false discovery rate (FDR) strongly depends in particular on the GC content and the average sequence identity of the input alignments. This is also the case for the fruit fly data (Additional file 1: Figure S2). To evaluate the influence of these two parameters we partitioned the set of repeat-filtered predictions into bins with narrow ranges of both GC content and sequence identity. We independently estimated the FDR for each subset (see “Methods” section for details). Requiring in addition a *pscore* >80, we observed that the resulting FDR estimates remained below 0.1 in most of the bins (Additional file 1: Figure S3), and predictions with a wide range of sequence identities were included in the remaining set (Additional file 1: Figure S2).

We observed a moderate increase of the FDR with GC content. Given the overall low GC content in drosophilid genomes, this fortunately does not constitute a substantial problem. It is also worth noting that CMfinder loses its power at sequence similarities below 40%: In this range, the FDR increased up to 0.5. Computing the FDR for bins depending on GC content, sequence identity, and *pscore* and using an FDR cutoff of 0.1, we retained 46,024 sequences for further analysis. 28% of these showed sequence identities below 60%, constituting promising CRSs candidates. Additional file 1: Figure S2 summarizes the number of CRS predictions as a function of FDR.

To see whether the sequence characteristics of the Rfam elements create a different error profile than seen globally, we re-analyzed a subset of the screen-wide FDR data, namely, the CMfinder results from the simulated MAF blocks containing the 527 Rfam elements summarized in Table 2. They yielded only 9 predictions (*pscore*>80); none corresponded to any of our 93 “positive” Rfam predictions. There were 76 predictions in the native alignments of those regions, yielding an estimated FDR <12*%*, in line with our global estimate. For details on FDR estimation we refer to the “Methods” section.

These initial CRS candidates were obtained from independent predictions on both strands. Owing to the near symmetry of RNA secondary structures, it is difficult to distinguish the reading direction of conserved RNA elements [39]. Furthermore, there is no reliable way to identify whether a single predicted element reflects a product from only one strand or if structured functional elements are produced by both strands. The latter has been described for the mir-iab-4 locus [40, 41]. Here, we made a conservative estimate by merging overlapping predictions on opposite strands, so that each genomic locus is assumed to produce one product. Since CMfinder searches for local structures and the available genome-wide alignments consist of many often very small blocks, we also merged adjacent elements that are separated by less than 30 nucleotides. This threshold is larger than the usual size of “holes” between consecutive alignment blocks but much smaller than the minimum distance between adjacent known ncRNAs, such as miRNAs in polycistronic clusters (Additional file 1: Figure S5). As a result, we estimated that 30,710 genomic loci encode conserved RNA structures.

### Annotation

Half of the predicted motifs were located in introns. This amounts to a slight enrichment compared to a uniform genomic distribution of CRS loci (Table 1). Introns often harbor ncRNAs that are processed from the host transcript [42]. In particular, several vertebrate snoRNAs are encoded in introns of ribosomal genes, allowing the snoRNA and the functionally closely related host gene to be co-expressed efficiently [43]. In addition, choice of splice sites and regulation of alternative splicing frequently involves secondary structures [44–47]. Hence, intronic CRSs constitute interesting candidates for structural elements of novel functional ncRNAs. Both UTRs of coding transcripts and the exonic parts of non-coding transcripts showed significant enrichments: 3.5*%* of the predictions fell into 5’-UTRs and roughly twice as many predictions in 3’-UTRs, representing a 1.5 and 1.6-fold enrichment, respectively, and the 661 loci (2.2*%*) in ncRNA exons constituted a 1.5-fold enrichment. In contrast, predicted motifs were under-represented in protein-coding exons (7.5*%*, 0.38-fold enrichment). This likely reflects the fact that coding exons are more conserved in the primary sequence than in their RNA secondary structure. About a quarter of the CRSs were found in intergenic regions and may belong to yet unknown transcripts.

**Table 1:** Overlap of CRSs with the *Drosophila melanogaster* genomic FlyBase annotation (dmel_r6.15, FB2017_02)

Feature	Number of CRSs overlapped	Percentage of CRSs overlapped	Fold enrichment	*P*-value	Total feature number	Number of features overlapped	Percentage of features overlapped
Exon coding	2294	7.5*%*	0.38	1.0	57906	2375	4.1*%*
Exon 5’-UTR	1082	3.5*%*	1.53	4·10^−12^	16930	1242	7.3*%*
Exon 3’-UTR	2409	7.8*%*	1.63	6·10^−113^	11288	1766	15.6*%*
Exon both UTRs	13	0%	1.13	0.72	415	16	3.9*%*
Exon ncRNA	661	2.2*%*	1.50	4·10^−15^	4120	588	14.3*%*
Intron	15565	50.7*%*	1.24	3·10^−221^	52410	6507	12.4*%*
Intergenic	8639	28.1*%*	1.12	5·10^−26^	12348	2924	23.7*%*
Unmapped alignment blocks	47	0.2*%*	−	−	1152	−	−

Annotation tracks were unified to avoid overlapping annotation elements and thereby ambiguous assignment of annotation categories to CRSs. In this context, annotation positions with overlapping 5’- and 3’-UTR exons have been collected in the “Exon both UTRs” category (see “Methods” for details). Predictions overlap the unified annotation feature by at least 1 nt, not considering strands. Prediction counts are given as rounded fractions according to the number of unified annotation features they overlap with. Percentages give the fraction of overlapping from total predictions. Fold enrichments and significance were calculated based on the annotation features contained in the CMfinder input alignments

The CMfinder predictions overlapped with 93 of the 527 ncRNAs annotated in Rfam and contained in the input alignments after repeat filtering (Table 2). This yields an estimated sensitivity of about 18% and an FDR of about 10%. We observed strong enrichments for miRNAs, H/ACA-box snoRNAs, composite snoRNAs (scaRNAs), snRNAs, as well as cis-regulatory elements. tRNAs showed moderate enrichment. Especially retroelements and stable intronic sequence RNAs (sisRNAs) as well as some tRNAs and H/ACA-box snoRNAs are located in short alignment blocks that had been removed prior to the CMfinder run and hence have been excluded as not contained in the input. rRNAs in addition often overlap repeats and also have been filtered out based on this criterion. The enrichment within the remaining lncRNA, C/D-box snoRNA and the histone 3’-UTR stem-loop annotations was not as strong as for other ncRNA classes, fitting the notion of these RNAs being less structured. Of the two ribozymes annotated in Drosophila, we recovered the nuclear Ribonuclease P (RNase P). Some of the predicted motifs may be associated with ncRNAs that are not completely contained in input alignment blocks and thus are not included in the list of known RNAs. The overlap thus is likely a bit higher than reported here. In any case, the overlap with Rfam is highly statistically significant overall, and in all but the smallest Rfam sub-categories (Table 2).

**Table 2:** Overlap of CRSs with the *Drosophila melanogaster* Rfam annotation (v.12.2)

Feature	Total feature number	Filtered feature number	Number of features overlapped	Percentage of filtered features overlapped	Fold enrichment	*P*-value	Number of CRSs overlapped
tRNA	294	247	32	12.9*%*	4.53	7·10^−30^	32
miRNA	92	85	18	21.1*%*	7.57	7·10^−28^	17
rRNA	156	6	1	16.6*%*	0	0.11	1
C/D-box snoRNA	45	41	2	4.8*%*	1.76	0.16	2
H/ACA-box snoRNA	27	14	4	28.5*%*	7.73	1·10^−5^	4
scaRNA	6	6	3	50.0*%*	18.04	6·10^−8^	3
snRNA	33	29	20	68.9*%*	21.15	2·10^−55^	20
lncRNA	15	15	2	13.3*%*	4.81	0.01	2
Cis-regulatory element	20	15	6	40.0*%*	12.03	8·10^−14^	7
Signal recognition particle RNA	4	4	0	0%	−	−	0
Histone 3’-UTR stem-loop	71	62	4	6.4*%*	2.33	0.03	4
Ribozyme	2	2	1	50.0*%*	18.04	0.04	1
Retroelements	121	1	0	0%	−	−	0
All	886	527	93	17.6*%*	5.89	2·10^−102^	93

Annotations with a base pair content of less than 30% were excluded. Predictions overlap the annotation feature by at least 50% of the prediction or the annotation feature size. *Filtered features* were filtered for features lying at least 50% of their size within the CMfinder input alignment blocks and overlapping a repeat by less than 50% of their size. The CMfinder input alignments did not contain sisRNAs, hence these are not listed here

### Overlap with other ncRNA screens

We compared the results of the CMfinder screen with previous surveys for drosophilid ncRNAs using EvoFold [48], REAPR [21], and RNAz [31, 49] in Table 3. The Sandmann RNAz data were filtered more stringently in order to identify specifically miRNAs and are therefore much more sparse than the predictions from the other screens. Considering only the less restricted screens using RNAz, EvoFold and CMfinder, the proportion of the overlaps is similar to what was observed using these tools in human [18, 50].

**Table 3:** Pairwise overlaps between predictions of the CMfinder and four additional screens for ncRNAs in drosophilids [21, 31, 48, 49]

	CMfinder	EvoFold	REAPR	RNAz(R)	RNAz(S)
CMfinder	**30710**	1618	3355	3967	410
EvoFold	1655	**22682**	2893	3583	331
REAPR	3340	2807	**30478**	19119	687
RNAz(R)	3993	3499	19358	**42479**	905
RNAz(S)	408	325	686	896	**2469**

*RNAz(R)* and *RNAz(S)* refer to the RNAz-based screens by Rose et al. [31] and Sandmann et al. [49], respectively. Given are the numbers of predictions in screen A (rows) that overlap predictions of screen B (columns) by at least 1 bp. Boldface values in the diagonal state the number of predictions in each dataset

The overlaps between surveys conducted with different methods are surprisingly small. However, assuming that the amount of sequence covered by predictions is small compared to the size of the genome, the expected overlap of two independent surveys of the same genome is the product of their sensitivities: 0.18×0.65=0.12 for our CMfinder screen and the Rose RNAz survey. However, both screens were performed using different genome releases, annotation versions and criteria with different FDRs. Therefore, the expected and the actual overlap between the screens are not directly comparable. A large overlap is observed only between the Rose RNAz screen and the REAPR predictions, which, however, are not independent of each other.

**Fig. 1:**
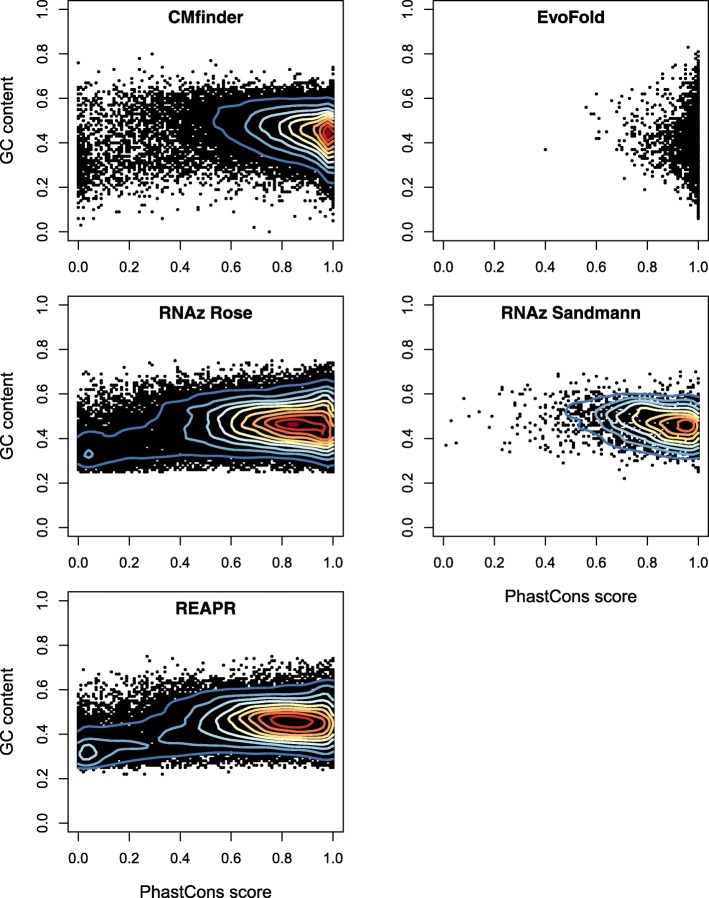
GC contents and sequence conservation measured in terms of phastCons scores [51] of the structured RNA predictions from CMfinder, EvoFold, REAPR, and RNAz (Rose, Sandmann) screens [21, 31, 48, 49]

Figure 1 shows that the CMfinder predictions are more similar to the RNAz predictions than to EvoFold data in terms of GC content and sequence conservation. The predictions of both methods cover a broad range of sequence conservation, while the phylogeny-based EvoFold method shows a strong preference for highly conserved predictions. However, alignment blocks with low sequence conservation are much less prevalent among the CMfinder predictions than among the RNAz predictions. An explanation for this difference can be inferred from a comparison of the situation in drosophilids to the one in vertebrates. In a genome-wide CMfinder screen in vertebrates [6], most of the predictions had a sequence identity between 60 and 70%, comparable with the drosophilid CMfinder predictions reported here (Additional file 1: Figure S1). However, the input alignments used in the vertebrate and drosophilid CMfinder screens differ greatly. In the vertebrate screen, only 10% of the input aligments overlapped annotated phastCons highly conserved elements [51]. Still, this small fraction of the input gave rise to 50% of all predicted CRSs [6]. In contrast, in fruitflies about 65% of the input alignments overlapped phastCons conserved elements. Hence it is not surprising that the vast majority of the Drosophila CRS predictions are located in highly conserved regions. The larger sequence variation in RNAz predictions might be explained by the higher false discovery rate of the tool. Specifically, the predictions with low phastCons scores may contain more false positives.

### Expression

To assess whether the predicted structures are likely to represent transcripts with real functions, we used expression data as a filter. Tissue and developmental stage-specific expression may be a good indication of biological function. We employed the modENCODE genome-wide tiling array dataset, which has a resolution of 38 bp, an exon expression score threshold of 300 (median of probe intensities for all probes found within that exon, normalized for cell lines), and consists of samples from 30 developmental stages and several Drosophila cell lines (both polyA+ and total RNA) [30, 52]. In the following, a CRS or genomic feature is categorized as expressed if it overlaps any tiling array transcript region by at least 50% of its size.

**Fig. 2:**
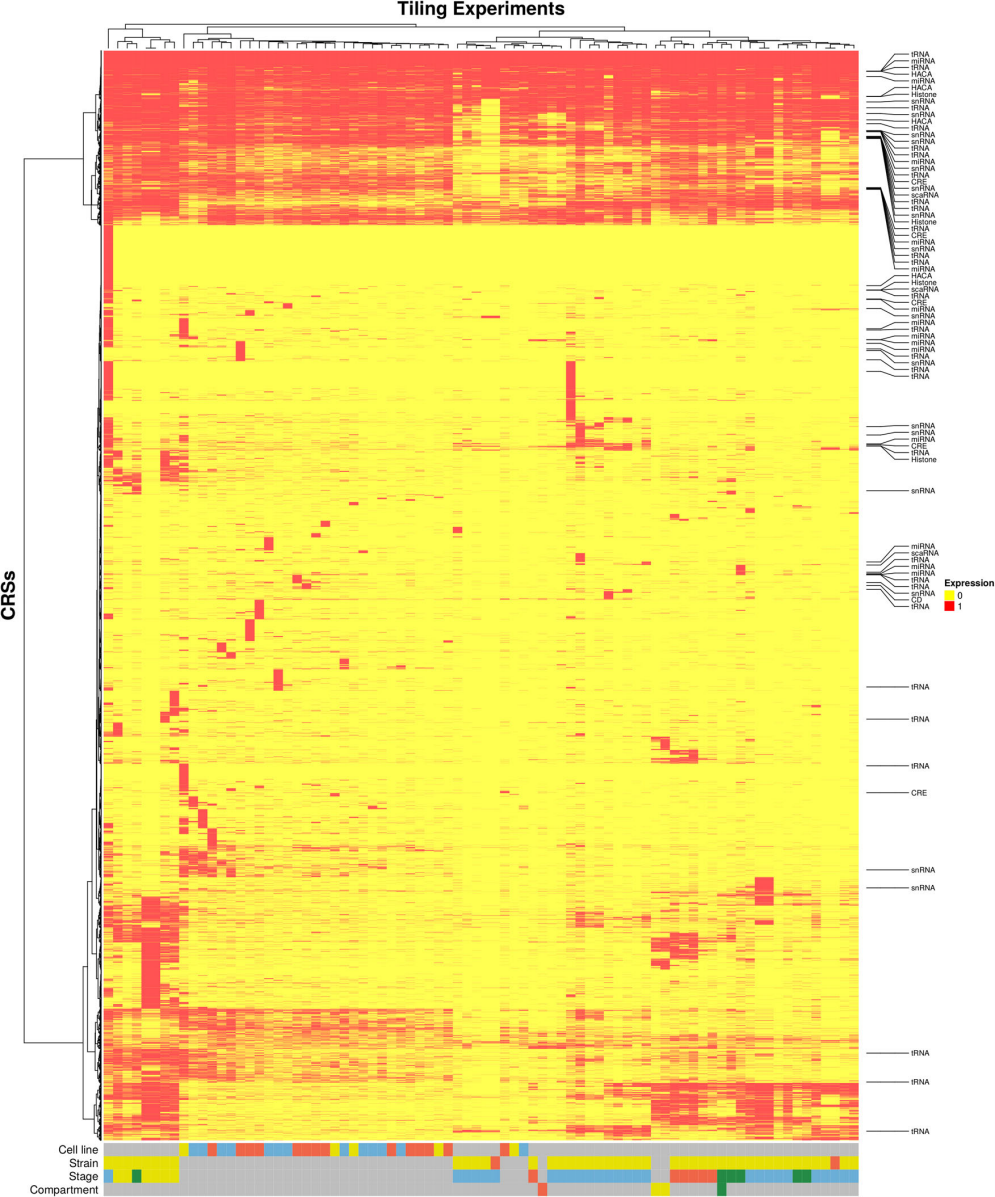
Expression of CRSs according to publicly available modENCODE tiling array experiments with an exon expression score threshold of 300 (median of probe intensities for all probes found within that exon, normalized for cell lines) [30, 52]. Only CRSs showing at least 50% overlap with at least one transcript region are considered. Non-coding annotations were obtained from Rfam (v.12.2). A subset of the samples is derived from cell lines, which almost form an individual, although heterogenous, cluster (first annotation line). All other samples are derived from flies of one of two different strains (second annotation line) and different developmental stages (third annotation line). Some of the fly samples are derived from specific compartments (fourth annotation line). The fly samples form much more homogenous clusters according to their stages and compartments. *Cell lines:* Blue: Embryo-derived. Red: Derived from prothoracic, mesothoracic, imaginal, antenna or eye-antenna disc. Yellow: Derived from central nervous system. *Strains:* Blue: Yellow cinnabar brown speck. Red: Oregon-R-modENCODE. *Stages:* Blue: Embryos. Green: Larvae. Yellow: Prepupae. Red: Adults. *Compartments:* Green: Gut. Red: Mated ovary. Yellow: Virgin Head

Of all CRSs expressed in at least one experiment (20,184), approximately a sixth showed expression throughout most stages and cell lines (Fig. 2). In contrast, the majority of CRSs are expressed in specific contexts. Expression patterns formed two clusters, separating cell line data from expression in flies. While developmental stages were not perfectly clustered together, there were some clear groupings: The six prepupal stages (yellow color in the stage annotation line) fell into an almost separate group. Five of the adult stages (red color in the stage annotation line) were grouped together, with similar CRS patterns in the adult female sample five days after eclosion and the mated ovary (see Additional file 1: Figure S6). The embryonic stages fell into several distinct clusters but were in general separate from other developmental stages (blue color in the stage annotation line).

CRSs that overlap annotated ncRNAs did not fall into obvious clusters with ncRNA classes. It is worth noting that annotated ncRNAs that overlap CRSs preferentially showed nearly ubiquitous expression. Indeed, well-expressed transcripts are expected to be found and annotated more easily than sparsely transcribed genes.

**Fig. 3:**
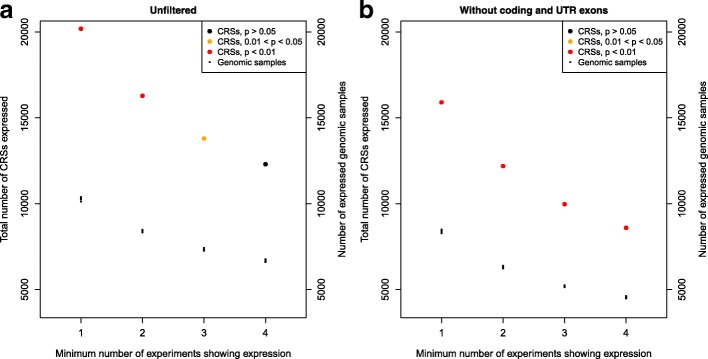
Significant enrichment for expression among CRS predictions. Expression is defined by a minimum number of modENCODE tiling experiments that show expression (x axis). As background we used 10 samples of randomly selected genomic loci of the same size and number as CRS predictions. The analysis was performed unfiltered (**a**) and filtered to exclude CRSs and genomic samples overlapping protein-coding and UTR exons (≥1 bp) to avoid mRNA exon bias (**b**). Significance of the enrichment of expressed CRSs is determined by the highest *p*-value from 10 samples calculated by Fisher’s exact test

Since CRSs are by definition expected to function at the level of RNA, we expect that CRSs are preferentially associated with expressed genomic regions. To test this hypothesis, we used the modENCODE tiling array data to assess the association of CRSs and expression in 100-bp windows sampled from the *D. melanogaster* genome. To avoid a bias due to the more abundant expression of protein coding loci, we removed all loci overlapping coding as well as UTR exons from the analysis. We did not exclude intronic loci, however, because intronic regions are often expressed as independent transcriptional units [53–56]. We observed a systematic enrichment of expression among CRS predictions (*p*<0.05, Fisher’s exact test). This result was independent of whether “expressed” was defined as a tiling array signal in a single experiment or whether a minimum number of positive tiling array data were required (Fig. 3).

#### Co-expression of intergenic CRSs and adjacent genes

Of particular interest are predictions of motifs for which there has been no functional evidence so far, i.e., in regions annotated as intergenic, but for which expression signals are observed. If a motif shows co-expression with its closest annotated gene, this might suggest a functional relationship. One possibility is that the predicted motif could be part of an incompletely annotated UTR. Alternatively it might reflect a novel transcript. As a measure of co-expression based on the modENCODE tiling array data we used a co-expression score that compares the number of tiling experiments in which a CMfinder prediction and its closest gene element (UTR or ncRNA exon) are expressed together or individually. The score *E*_*co*_, see Eq. (1) (see “Methods” section), is the difference between the following two ratios: The number of experiments in which both CRS and closest gene (independent of the genomic distance) are expressed normalized by the total number of experiments with CRS expression (Ratio 1); and the number of experiments where the closest gene but not the CRS is expressed normalized by the total number of experiments without CRS expression (Ratio 2). With the help of this score we can determine whether co-expression suggested by the tiling array data is positive or negative. For perfect positive co-expression, Ratio 1 equals 1 (the CRS is exclusively expressed together with its closest gene element), and Ratio 2 equals 0 (the gene element is never expressed without the respective CRS). As a consequence, the difference of both ratios is 1. For negative co-expression the situation is the converse, resulting in a co-expression score of −1. To raise the reliability of the scores, only CRSs that are expressed in at least 3 tiling experiments were considered here.

**Fig. 4:**
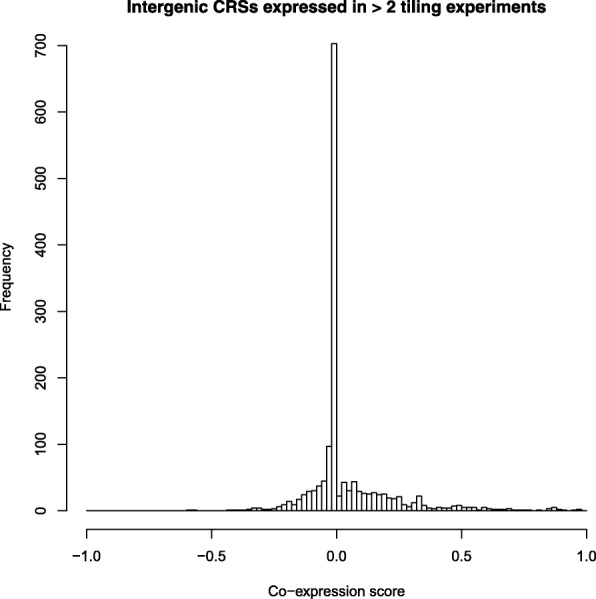
Co-expression of intergenic CRSs with their closest annotated gene element according to modENCODE tiling array experiments [30]. A co-expression score of 1 indicates perfect positive co-expression, i.e. both CRS and closest gene are exclusively expressed together, −1 indicates perfect negative co-expression, i.e. both elements are only expressed individually, and a score of 0 indicates that the expression of CRS and closest gene were observed to be independent of each other

The majority of CRSs showed a co-expression score of exactly 0, indicating that their expression was not related to that of their closest gene elements (Fig. 4). Distinguishing co-expression signals from noise is a challenge especially for co-expression scores close to 0. In theory, we would assume functional positive co-expression only at a perfect co-expression score of 1 since the adjacent gene can only be expressed if the activating CRS is present as well. However, due to known biases of tiling arrays against sequences with low GC contents and very stable secondary structures [18, 50] we cannot expect complete detection of all expressed transcripts. Therefore, we empirically chose score cutoffs of ≥0.5 and ≤−0.5 for positive and negative co-expression, respectively (also see Additional file 1: Figure S7). While 55 out of 1540 CRSs had scores ≥0.5, negative co-expression was observed rarely: Only two CRSs had a co-expression score below −0.5. All CRSs are available at https://rth.dk/resources/rnannotator/crs/insect/.

Most CRSs with relevant co-expression scores are expressed under few conditions. Of the 57 CRSs meeting our co-expression criteria, only 15 are expressed under more than five conditions. This is in agreement with the fact that most of the CRSs are expressed in specific contexts. One CRS (see “Examples of novel structures” section) was expressed in 29 experiments and in all of these together with its closest gene at a distance of only 12 bp of the annotated 5’-UTR (in case CRS and the closest gene element overlap 100% with tiling array transcript regions, instead of 50% as applied during the co-expression analysis). This strongly suggests that the current UTR annotation is incomplete and the CRS is in fact a structured UTR element.

As our assessment of co-expression is based on adjacency, it is conceivable that the physical order of co-expressed CRS and closest gene is also preserved in other species. We therefore compared the genes that are adjacent to CRSs in *D. melanogaster* and in other species present in the CRS alignments. Of the 11 currently annotated non-*melanogaster* drosophilid species (FlyBase release FB2018_04), two thirds were required to fulfil the respective synteny criteria in the following analyses. For 13 of the 57 CRSs co-expressed in *D. melanogaster*, the closest genes in the other species were the orthologs of the closest *D. melanogaster* gene. However, this number is likely too conservative as we cannot expect all annotations to be complete and all orthologous relationships between genes of different species to be resolved entirely. More importantly, especially in more distant relatives of *D. melanogaster*, genes can be inserted between the CRS and the ortholog of the *D. melanogaster* gene. Hence, we also considered more relaxed criteria to define synteny: Looking for the ortholog of the *D. melanogaster* gene in each species, regardless of its distance from the CRS, we found 32 CRS-gene pairs to be in the same orientation in other species as in *D. melanogaster*, i.e. both the *D. melanogaster* gene and its ortholog were located either upstream or downstream of the CRS. When we applied an empirical maximal distance of CRS and closest *D. melanogaster* gene (or its ortholog) of 20,000 bp (see Additional file 1: Figure S8), still 18 of these 32 CRS-gene pairs passed the synteny filter. Finally, assuming that phylogenetic distance and quality of the annotation vary between species, we compared the closest genes in *D. melanogaster* and other species in a pairwise manner. In the species most closely related to *D. melanogaster*, *D. simulans* and *D. sechellia*, the genes neighboring 25 and 26 CRSs were orthologs of the gene closest to the CRS in *D. melanogaster*, respectively. The syntenic relationships of a subset of co-expressed CRS-gene pairs in *D. melanogaster* and their orthologous counterparts in other drosophilids provide another level of evidence for functionality of these CRSs.

#### Developmental stage and cell line specific expression of CRS-containing biotypes

**Fig. 5:**
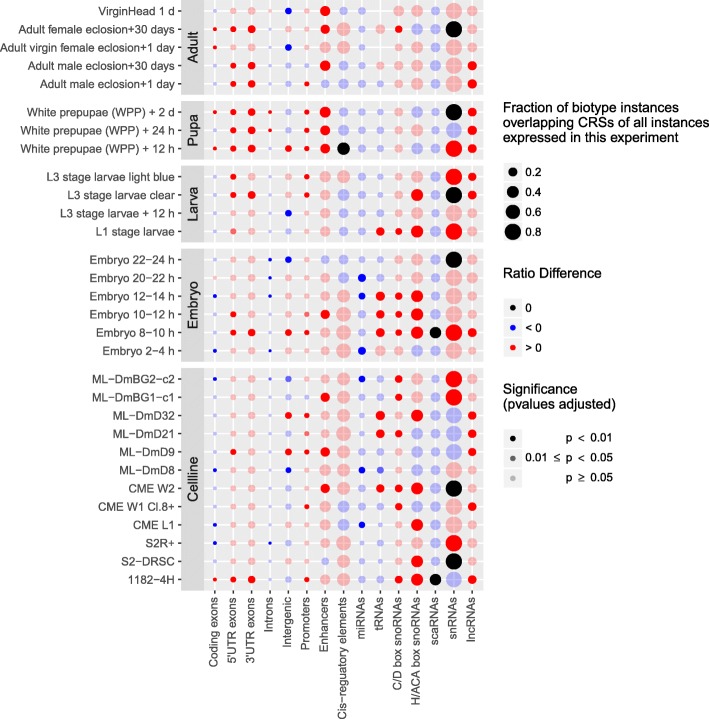
Over- and underrepresentation of biotype instances with CRSs compared to instances without CRSs (‘ratio difference’, color coded) in *Drosophila melanogaster* developmental stages and cell lines. Only instances expressed in at least three tiling array experiments and contained in the CMfinder input alignments by at least 50% of the feature size were considered here. Tests of significance (indicated by opacity) assess whether biotype instances with CRSs are expressed more often in a particular stage compared to all other stages than expected by chance. *p*-values have been adjusted for multiple hypothesis testing (Bonferroni). The statistical test has been performed for all stages and cell lines, but in the interest of visibility, a representative subset of stages and cell lines of only total RNA samples has been chosen for this figure. For the full version of the plot, see Additional file 1: Figure S9

*D. melanogaster* development is regulated by an orchestra of specific genes, see [57] and the references therein. Here, we connect the expression patterns of CRSs across developmental stages and cell types as a first step towards elucidating their potential roles in fruitfly development. For this as well as the following analysis, we associated genomic locations with a “biotype”, i.e., a class of RNAs defined by similar functional and/or structural characteristics, such as miRNA, C/D box snoRNA, or 3’-UTR exon. We asked if expression of CRSs belonging to a particular biotype was statistically over- or underrepresented in a particular developmental stage or cell line (Fig. 5). In order to achieve a fair comparison we normalized the number of instances of a biotype expressed in a particular stage by the number of instances of the same biotype expressed in any of the other stages. For each biotype we then calculated the difference of these ratios for the subsets with CRSs (*R*_*CRS*_) and without CRSs (*R*_¬*CRS*_), see Eq. (2) (see “Methods”). If this difference is positive, there are more instances with CRSs expressed in this stage compared to other stages than is the case for instances without CRSs. A significant difference between *R*_*CRS*_ and *R*_¬*CRS*_ may indicate a general role of the CRS-containing biotype instances in differentiating this stage. See Methods for more details of the analysis.

Not surprisingly, CRSs detected by our screen were particularly abundant for ncRNA classes, i.e., the biotypes H/ACA box snoRNAs, scaRNAs, snRNAs, and for cis-regulatory elements. CRSs were relatively rare in highly abundant biotypes such as introns, intergenic and exonic regions. Patterns of stage-specific over- and underrepresentation of CRS-containing biotypes were more similar to each other between pupae and adult stages, and more homogeneous than for the embryonic and larval stages (also see Additional file 1: Figure S9). Cell lines showed different patterns of enrichment and underrepresentation than developmental stages. The pupae stages formed the group with the statistically most significant enrichments of CRS-containing biotypes. Among these were mainly CRSs in 5’- and 3’-UTR exons, introns, promoters, enhancers, and lncRNAs. Adult stages shared some of these enrichments, but also exhibited an underrepresentation of expressed CRSs in intergenic regions. Larval and embryonic stages differed from the other stages in that there were fewer stages enriched for CRS-containing UTR exons and lncRNAs and an even stronger underrepresentation of CRS-containing instances of several biotypes, e.g., introns and miRNAs. However, especially H/ACA box snoRNAs with CRSs were enriched in a number of embryonic and larval stages.

In cell lines we observed expression enrichment of CRS-containing UTRs less frequently than in any group of developmental stages. In contrast, ncRNA biotypes, especially snoRNAs, tRNAs, and intergenic regions were more often enriched with CRSs (also see Additional file 1: Figure S9). In summary, CRSs appear to be part of expression patterns that distinguish individual developmental stages from others.

#### Differential expression of CRSs

**Fig. 6:**
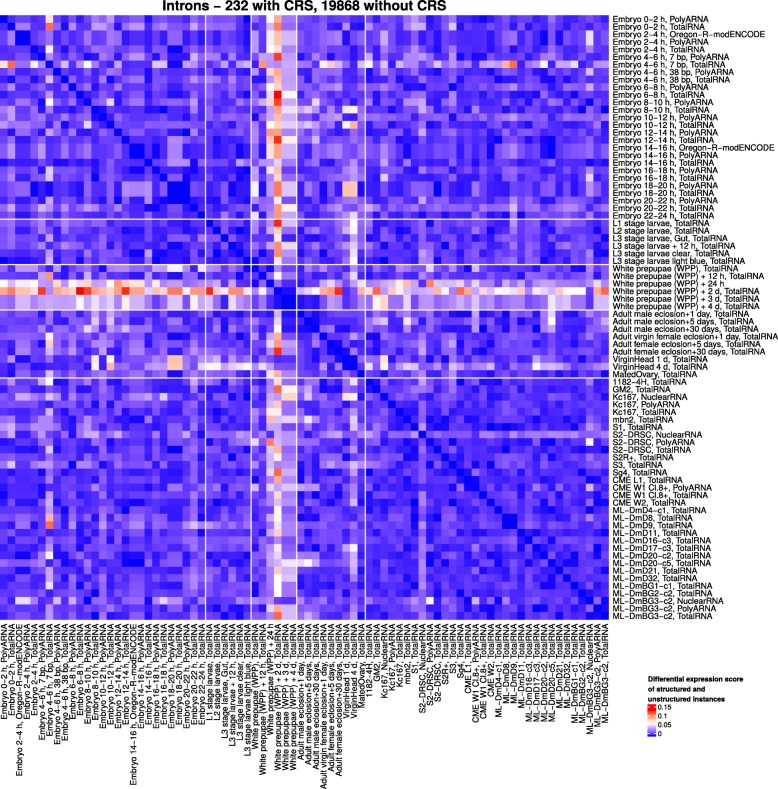
Pairwise differential expression scores for introns contained in the CMfinder input alignments and expressed in at least three developmental stages or cell lines. The score compares the differential expression of introns containing CRSs and introns without CRSs. The higher the score on a scale of 0 to 1, the more structured introns and the less unstructured introns are differentially expressed

In order to elucidate the functional potential of CRSs in development in more detail, we aimed to identify pairs of developmental stages for which CRSs exhibit differential expression correlated with other biotype instances. We calculated a differential expression score *E*_diff_(*i*,*j*) as defined in Eq. (3) (see Methods) for each pairwise combination of modENCODE experiments *i* and *j* that compares the differential expression of CRS-containing instances and instances without CRSs (Fig. 6). The score can take on values from 0 to 1. The maximal score of 1 means that all structured instances are differentially expressed between experiments *i* and *j* whereas none of the unstructured instances is. The product in the equation gives higher impact to situations with high differential expression of structured instances. See Methods for more details of the analysis.

For most biotypes, *E*_diff_ was small, i.e., the overall expression pattern did not differ much between individual stages. However, there were some structured intronic regions that were differentially expressed between white prepupae and most of the other stages and also cell lines. This could also be explained by the differential expression of the corresponding gene. Hence, in the next step we specifically considered differentially expressed introns in genes of which no exon is expressed in the same experiment. In Fig. 6, one of the most prominent red areas with high differential expression scores exists between intronic loci of white prepupae (two days) and the 12-14 h embryonic stage (both total RNA samples). Of these differentially expressed introns with CRSs, 29 were directly flanked by exons that were not expressed under the same conditions, and 3 were contained in genes of which not a single exon was expressed in the same experiment. One of the overlapping CRSs is referred to in more detail in the “Examples of novel structures” section. This observation suggests that the CRSs could be transcribed independently of the host genes. We note that the differential expression scores in this analysis did not rise above 0.15 for introns. Intronic loci with large differential expression thus are interesting candidates for novel functional transcripts.

### Examples of novel structures

**Fig. 7:**
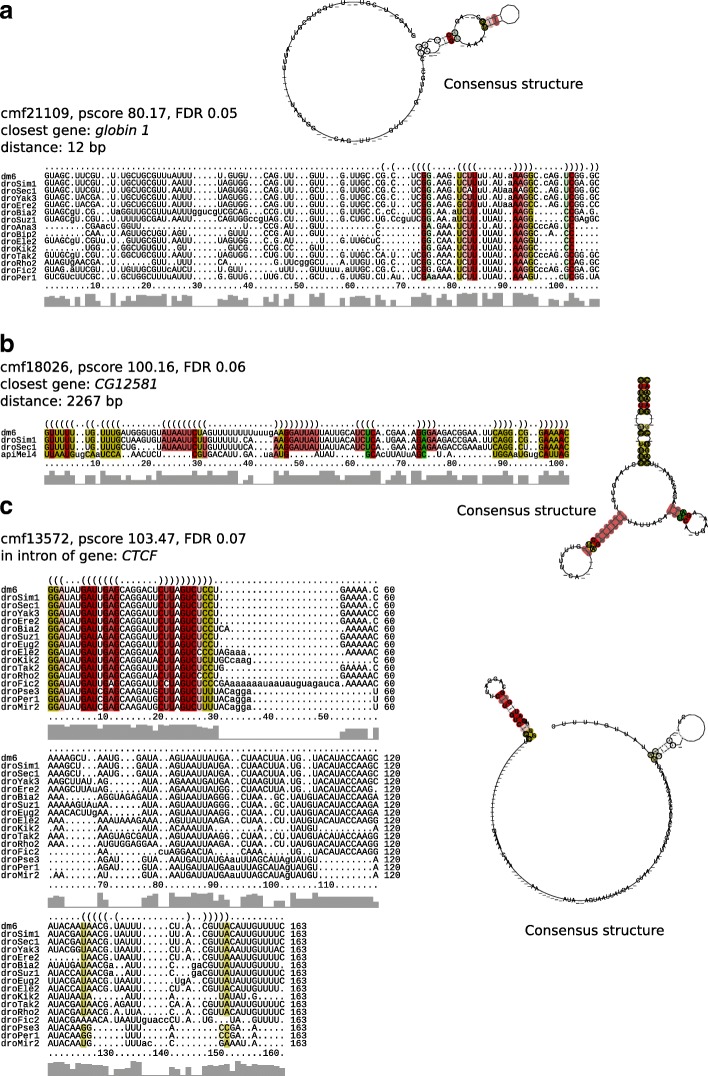
Examples of CMfinder predictions that are part of putative novel transcripts or possibly incomplete annotations. Example **a** with a particularly high co-expression score and small distance to the closest annotated gene could be part of an incompletely annotated UTR. Example **b** is located much further from the closest annotated gene and hence could be part of a putative novel independent transcript. Example **c** is located in a differentially expressed intron of a gene of which no exons are expressed in the same developmental stage. For more details see description in the main text. Alignment and secondary structure visualization were performed using RNAalifold [79, 80]

Based on the co-expression and differential expression analysis, a number of not previously annotated interesting structured RNA candidates were identified in intergenic or intronic regions. We present three examples representing different kinds of functional evidence: Positive or negative co-expression with the closest annotated gene, very small genomic distance to an annotated UTR, location in an intron showing differing expression from the adjacent exons, and a stable and complex secondary structure. Prediction DC0021109 (Fig. 7a) shows a perfect positive co-expression score with its closest gene *globin 1*, i.e., neither CRS nor *globin 1* are expressed alone in any of the 29 experiments in which expression was observed in this case (in case DC0021109 and the closest exon of *globin 1* overlap 100% with tiling array transcript regions, instead of 50% as applied during the co-expression analysis above). Since the genomic distance between them is only 12 bp, the annotated UTR of *globin 1* is most likely incomplete and DC0021109 is a structured UTR element. An example with a negative co-expression score of −0.57 is DC0018026 (Fig. 7b) with its closest gene *CG12581*, encoding a mostly unknown protein with a phospho-tyrosine binding domain, which may be involved in a wide range of processes like neural development, tissue homeostasis or cell growth [58]. DC0018026 folds into a compact, stable consensus structure (*ΔG*=−15.99 kcal/mol) comprising a multi-branch loop with two hairpins and an external stem. In contrast, DC0013572 (Fig. 7c) is located in an intron of the zinc finger transcription factor gene *CTCF*, which is involved in chromatin organization [59]. The secondary structure of the CRS features two hairpins with a longer conserved single-stranded stretch in between.

## Discussion

We conducted a study of evolutionarily conserved RNA structure (CRS) elements in the *D. melanogaster* genome that was designed to assay genomic regions that are only loosely constrained at the sequence level. Therefore, we employed CMfinder to leverage structural alignments. Although CMfinder can detect CRSs also in highly sequence-conserved regions (unless the sequence identity reaches 100%) we observed that its sensitivity was limited when conservation at sequence level was high. As a consequence, the recall on well-studied RNA classes such as tRNAs, miRNAs and snoRNAs, all of which are very conserved at sequence level, was only moderate. These classes were readily detected in an earlier RNAz screen which operated on sequence-based alignments [31].

While RNAz performs best in the vicinity of 80% average pairwise sequence identity [31, 50], the majority of the CMfinder predictions were observed to lie between 60 and 70% in the screen on vertebrate genomes, and more than one third showed sequence identities below 60% [6]. However, a similar assessment for CMfinder on insect genomic alignments has been missing so far. As with RNAz, the number of CMfinder predictions decreased with sequence similarity. However, the predictions from the present screen have a much smaller FDR than previous RNAz results (less than 10% for CMfinder compared to up to 50% for RNAz [50]). The increased accuracy is ensured by using different cut-offs in different ranges of GC content and sequence conservation, thus controlling the FDR approximately independently of these parameters (Additional file 1: Figure S3). Nevertheless, we find a comparable number of CRSs. Although the overall sensitivity of the CMfinder screen was only moderate, it targets CRSs in a different range of conservation than other tools, emphasizing the usefulness of the CMfinder approach, in particular to screen in the low conservation range. At present, no single tool is capable of uncovering the entire wealth of RNA structure that is under selective constraints. This calls for research into improved methods for identifying selection pressures on RNA structure that can capitalize on the increasing amount of genome data that are becoming available for comparative genomics approaches.

Comparing the CRSs with genome-wide expression data from a broad range of developmental stages and cell lines, we found that in addition to a large number of nearly ubiquitously expressed loci, there were also sizable groups expressed in specific developmental stages or cell lines. The most statistically significant enrichment of expressed CRSs was observed in pupae, mainly located in UTR exons, promotors, enhancers, and lncRNAs. Interestingly, cell lines express different sets of CRSs than native developmental stages. This is in accordance with the respective modENCODE study of Drosophila cell lines [52]. Only a small fraction of intergenic CRSs was found to be co-expressed with the adjacent protein coding genes, indicating that most intergenic CRSs are independent genetic units.

An unexpected finding from our analysis was the differential association of *detected* CRSs with development type in biotypes such as snoRNAs and miRNAs, which are known to be conserved in both sequence and structure. This implies that there are differences in the patterns of sequence and/or structural evolution of these ncRNAs that are strong enough to affect how well they are detected by CMfinder. A characterization of these differences will require a detailed investigation into possible subclasses of miRNAs and snoRNAs as well as a comparative study of species outside the drosophilid clade – and hence goes well beyond the scope of the present contribution.

One third of the predicted CRSs were not expressed, according to tiling array expression data. Some of this can be explained by the expected moderate fraction of false positive predictions. However, tiling arrays have several intrinsic biases that may prevent them from measuring CRSs: First, there is a bias against CRS sequences with low GC contents, since these interactions are less stable, and finding optimal stringency parameters to remove randomly bound RNAs but retain all true positives is challenging [18]. Furthermore, very stable secondary structures may not be captured since intramolecular folding will compete effectively with hybridization to the array [50]. Finally, also biological factors are likely to have an impact: Expression of transcripts that are expressed at very specific time points in development can easily be missed if there was no sample taken at exactly this time point. Also, differing external conditions might be necessary to induce expression of specific transcripts which cannot all be covered by any large-scale screen.

As it was observed in the mammalian CRS screen that CRSs hold the potential to bind RNA binding proteins (RBPs) [6], we assume that this is also the case for the fly genomes. Although some studies suggest that some RBPs appear to prefer single-stranded regions [60], other studies suggest that most RBPs prefer structured RNA, such as Staufen [61], Roquin [62] or MLE [63]. Computational surveys [64, 65] strongly suggest that structured binding sites are by no means rare. The interpretation of many CRSs as conserved RBP binding sites not only provides a biologically plausible explanation for the large number of detected loci in otherwise poorly conserved regions, but also suggests that it will be worth while (a) to engage in a large scale clustering of the predicted elements and (b) to compare the detected CRSs also across large phylogenetic distances, in particular with the elements reported in mammals [6, 32].

While the ultimate goal is to understand the role of conserved RNA structures in development, the computational survey reported here has to be content with providing starting points for following research. Our data show that there is a large set of CRSs with specific expression patterns that suggest their involvement in development and differentiation. Of course, such correlational data cannot distinguish between causal regulators and downstream consequences, but they narrow the list of candidates for further studies, both regarding cis-regulatory motifs and presumably independent ncRNA transcripts.

Although beyond the scope of this contribution, it will be relevant to characterize the stability of structures further within their respective biotypes. For example, 75% of the CRSs are located in introns or intergenic regions and can probably be further sub-categorized both by their stability and structural similarity using clustering techniques [66, 67]. It would be interesting to know whether CRSs found in other biotypes show patterns depending on the type of RNA they are part of.

## Conclusion

Currently there are approximately 700 structured ncRNAs known in fruitflies [68], as well as thousands of unstructured ncRNAs (mostly lncRNAs [25–27]). While unstructured RNAs are generally easier to identify based on a certain level of sequence conservation, functional RNA structures are more hidden, and dealing with conservation on a structural level requires more elaborate and computationally expensive approaches. Hence, the true number of ncRNAs, especially of structured ones, is expected to be larger than the set we currently know. Accordingly, we found a large number of structurally conserved putative candidates in intergenic and intronic regions, many of which are likely to be functional according to evidence from expression analyses.

Due to the strong tendency of most RNAs, be they functional or not, to take on secondary structures, computational screens for CRSs need to deal with a certain trade-off between sensitivity and specificity as well as rather high false discovery rates, although we believe the latter to be lowered considerably in CMfinder screens. As a consequence, different tools for the prediction of conserved RNA structures yield only moderate overlaps when applied to the same genome. Screens conducted with alternative methods on previously investigated genomes therefore are a useful endeavor that contributes complementary data. In conclusion, our study has substantially expanded the repertoire of conserved RNA structures in fly genomes and in contrast to previous studies uncovered CRSs within the context of expression throughout all developmental stages and many cell lines.

## Methods

### Computational screen for CRSs

The 27-way MULTIZ [69] alignment consisting of 23 drosophilids and four additional insect species was downloaded from the UCSC Genome Browser (*Drosophila melanogaster* genome dm6, Aug. 2014, BDGP Release 6 [70]). The MAF (multiple alignment format) files contain sequences for chromosome arms 2L, 2R, 3L, 3R and chromosomes 4, X, Y, as well as mitochondrial sequences (M) and sequences in unassembled scaffolds. MAF blocks containing fewer than three sequences or that are shorter than 50 bp were removed. For all remaining MAF blocks, the reverse complement was generated in addition to be able to make predictions on both strands. Gaps were removed from the alignments and the sequences were fed in their unaligned form into CMfinder.

We ran CMfinder (version 0.2.1) with default settings separately on the forward and the reverse strand of the native genome alignment. Default settings are as follows: The maximum number of candidates predicted in each sequence (i.e. MAF block) is 40. At most, 5 single stem-loop motifs with a base pair span between 30 and 100 bp and 5 double stem-loop motifs with a base pair span between 40 and 100 bp are returned. Motifs on the same strand are merged by CMfinder if the predicted structure is consistent in both overlapping motifs. The prior for the expected fraction of sequences containing the motif is 0.8. The CMfinder-specific *pscore* [35], (CMfinder version 0.2.2) was computed for all predicted motifs. It is fundamentally similar to a general time reversible model of sequence evolution extended to include both single-stranded and base-paired regions. Some model parameters were trained using vertebrate Rfam alignments, but we scored our candidate motifs with respect to a phylogenetic tree having topology and branch lengths as estimated for drosophilids (dm6.27way.nh from ref. [70]; CMfinder’s -t option). As shown in Additional file 1: Figure S2, a good *pscore* is well-correlated with lower estimated FDR across the spectrum of sequence identity and GC content. Sequence identity and GC content were calculated for all CMfinder output alignments. As a reference sequence for all predictions we used our species of interest, *Drosophila melanogaster*, and therefore only considered predictions containing this species. All genome coordinates used in the following were derived from the reference genome.

### Background model

We estimated the false-discovery rate among the CMfinder predictions by synthesizing “background” alignments using SISSIz (version 2.0 [16]). Specifically, for each input MAF block, one companion randomized alignment was produced using SISSIz with the following options: --simulate --tstv --maf -n 1. This simulates sequence evolution from an ancestral sequence derived from the given MAF block using an evolutionary model that preserves mono- and di-nucleotide frequencies in expectation, while exactly preserving the input’s gap- and local conservation patterns. Transition and transversion rates are estimated from the input data (--tstv), and one random alignment in MAF format is generated per input (--maf -n 1). CMfinder was run on both strands of the shuffled genome alignment in the same way as on the native alignment.

### False discovery rate

In order to find a threshold to filter out the most unreliable predictions, *pscore* lower boundaries from 50 to 150 were applied and the distributions for *pscore*, minimum free energy, GC content, sequence identity, length and number of species of the motif alignments as well as the number of predictions remaining were visually inspected. Based on this, we applied a *pscore* cutoff of *p*>50 to reduce the number of predictions to a manageable amount.

All predicted motifs with a *pscore* >50 were filtered for overlap with annotated repeats (as provided through the UCSC genome browser [71]) using bedtools intersect [72], removing all predictions that overlap a repeat by at least 50%.

To estimate the false discovery rate (FDR), GC content and sequence identity were categorized so that each bin comprises comparable numbers of predicted motifs with these features. CMfinder input MAF blocks were categorized into the same bins. Since their number is sufficiently high in each bin (i.e. more than 100 MAF blocks), all bins were considered, even in case the numbers of predictions in a bin were low. The FDR was estimated for all motifs in each bin with a particular *pscore* cutoff as

$$\begin{aligned} \text{FDR estimate} &= \frac{\text{False Positives}}{\text{False Positives} + \text{True Positives}}\\ & = \frac{\text{\# predictions on the shuffled alignment}}{\text{\# predictions on the native alignment}} \\ \end{aligned} $$

Based on the number of predictions left, the individual FDR heatmaps and relationship between mean FDR and sequence identity depending on pscore cutoff, cutoffs of both *pscore*>80 and FDR ≤0.1 were taken to filter out the most unreliable predictions. FDR estimates were assigned to each motif according to the FDR of the respective bin and pscore cutoff.

### Annotation

For the overlap with the existing annotation, the most recent *Drosophila melanogaster* annotation data for the dm6 genome release were obtained from FlyBase (dmel_r6.15, FlyBase release FB2017_02) for the genomic annotation (exon type, intron or intergenic region) [29] and from Rfam 12.2 for the non-coding RNA annotation [68].

When converting the FlyBase annotation fasta files into bed files, split entries were converted to a single bed entry without considering splicing. In the rare cases of genes derived from both strands such as trans-spliced mod(mdg4) [73], separate entries were created for both strands.

In order to annotate each prediction unambiguously, the FlyBase genomic annotation tracks were unified such that each nucleotide has only one annotation category assigned. For this purpose, all annotated coding sequences as well as genes were merged using bedtools merge. Each annotated exonic region was categorized either as coding exon if located within coding sequence boundaries or otherwise as non-coding exonic region. Drosophilid Rfam annotations were added to the non-coding exonic regions. To determine if a non-coding region belongs to a ncRNA or to a UTR, each exon’s gene parent was checked for the presence of a coding sequence. In case ncRNA and UTR exons overlap (this was observed for approximately 5% of all UTR exons), the ncRNA-exonic character was prioritized and the region of overlap annotated as ncRNA-exonic region. The regions in which 5’- and 3’-UTR exons overlap are categorized as exons of both UTRs because in this case no meaningful prioritization of one UTR type over the other can be made. Then, each so generated annotation bed file was merged using bedtools merge and all resulting exons were subtracted from the list of all merged genes to obtain all introns. All exons and introns were subtracted from the complete genomic sequence to obtain all intergenic regions.

For the annotation and all subsequent analyses (unless explicitely mentioned otherwise), individual predictions were merged strand-independently up to a distance of 30 nt using bedtools merge.

For the genomic annotation, we counted how many annotation elements (individual exonic, intronic or intergenic regions) overlap a given prediction (≥1 bp) without considering strands and then assigned the respective fraction, i.e. 0.5 in case a prediction overlaps two genomic classes. This approach was chosen because the unified exons and introns can be very short. Therefore, a CRS might overlap a number of different categories, and an overlap of at least 50% of the CRS size is less meaningful in these cases.

For the overlap with the non-coding annotation, only ncRNAs lying by at least 50% of their size within individual CMfinder input MAF blocks were considered since annotated structures that are not covered by the alignment cannot be predicted. The minimum overlap of 50% takes into account that many ncRNAs consist of several shorter structured motifs, which still can be predicted by CMfinder even if only a part of the complete sequence is contained within a MAF block. We only included Rfam annotations with a minimum base pair content of 30%, which means at least 30% of the positions of a sequence must be involved in base pairing. In order to identify known ncRNA elements covered by the predictions, CMfinder predictions and Rfam annotation were intersected using bedtools intersect with a minimal overlap size of at least 50% of the prediction or the annotation element size.

For each intersection of CMfinder predictions and genomic or ncRNA annotation, the fold enrichment FE was calculated as

$$\text{FE} = \frac{\frac{\text{\# merged overlapping queries}}{\text{\# queries}}}{\frac{\text{target size (nt)}}{\text{background size (nt)}}} $$

The background size is computed as the total number of columns in the CMfinder input MAF blocks. The target size is defined as the total size of all annotation elements under consideration that overlap a MAF block by at least 1 nt. The significance of each enrichment was calculated using the pnorm function in R as previously described [18]. Specifically, the number of observations was the number of overlaps and the mean was calculated as the product of the total number of CRS candidates and the fraction of the input covered by the annotation.

The FDRs for the recovered and not recovered fractions of the Rfam annotation were estimated in a similar manner as the genome-wide FDR, but only including individual predictions (*pscore*>80, repeat-filtered) from native and shuffled alignments that overlap (recovered or not recovered) Rfam annotations, without considering strand, GC content, or sequence identity.

### Comparison with other ncRNA screens

We compare our predictions to four other genome-wide screens for ncRNAs in drosophilids: 42,482 predictions from an RNAz screen [31], 2469 predictions from a more restrictive RNAz screen aimed at finding miRNAs [49], 30,478 predictions from a REAPR screen [21], and 22,682 predictions from an EvoFold screen [48]. Site-specific phastCons scores based on the MULTIZ 27-way insect alignment were averaged for each predicted motif or CRS, respectively. GC contents were calculated for each *D. melanogaster* sequence. For the prediction overlaps, the coordinates of all three screens were transformed from dm2 to dm6 genome release using the UCSC LiftOver utility (https://genome.ucsc.edu/cgi-bin/hgLiftOver). Predictions were intersected using bedtools intersect with a minimal overlap of 1 bp.

### Expression data set

Tiling array data were obtained from the modENCODE database (v32 [74]), comprising 3,665,935 transcript regions. Each of the 80 experiments corresponds to expression in one cell line or in one developmental stage of one of two fly strains, either total, polyA, or nuclear RNA-sequenced. In a minority of the experiments expression was evaluated specifically in virgin heads, mated ovaries, or the larval gut. Throughout this study, if not stated otherwise, a CRS or any annotation instance is defined as expressed if it overlaps a merged transcript region by at least 50% of its size. For the expression heatmaps, only CRSs showing expression in at least one experiment were considered. The non-coding RNA annotation was obtained from Rfam (v.12.2).

### Expression enrichment

To obtain a random background for Fisher’s exact test, the *D. melanogaster* genome was divided into 100-bp windows (approximately the average size of a CRS), and 20,184 of the windows were sampled randomly. Samples expressed in at least one to four modENCODE experiments were intersected with the CRSs (overlap at least 50% of the CRS or genomic sample size). The resulting contingency table for each minimum number of experiments consists of the numbers of genomic samples expressed and not expressed and these overlapping CRSs or not. Sampling and Fisher’s exact test were carried out 10 times for each minimum number of experiments. In a second test, windows and CRSs overlapping coding or UTR exons were removed in order to avoid a potential mRNA exon bias. Sampling and Fisher’s exact test were carried out as previously.

### Co-expression with adjacent genes

To evaluate co-expression of a prediction with its closest gene element we define the co-expression score *E*_*co*_ as

1 $$  E_{co} = \frac{E_{cg}}{E_{c}} - \frac{E_{g \neg c}}{E_{\neg c}},  $$

where *E*_*cg*_ is the number of experiments in which both the CRS and its closest gene element are expressed, *E*_*c*_ is the number of experiments in which the CRS is expressed, *E*_*g*¬*c*_ is the number of experiments in which the closest gene element is expressed but not the respective CRS, and *E*_¬*c*_ is the number of experiments in which the CRS is not expressed. $\frac {E_{cg}}{E_{c}}$ is also referred to as Ratio 1 and $\frac {E_{g \neg c}}{E_{\neg c}}$ as Ratio 2.

For the analysis of synteny of co-expressed CRS-gene pairs, orthologs of *D. melanogaster* genes in all 11 annotated non-*melanogaster* species [75] were obtained from FlyBase (FlyBase release FB2014_06, the most recent release with all genome releases corresponding to the genome releases used in the MULTIZ alignment), as well as the corresponding gene annotations for each species. Where necessary, FlyBase chromosome/scaffold identifiers were transformed into UCSC identifiers with the help of the respective assembly reports and GenBank accession numbers [76]. In case of ties when determining neighboring genes of CRSs, i.e., multiple genes with the same distance to the CRS in *D. melanogaster* or any other species, at least one ortholog had to fulfil the respective synteny criterion (being the ortholog to a *D. melanogaster* closest gene, being in the correct orientation with respect to the CRS, or being within the maximum distance, 20,000 bp, of the CRS).

### Experiment-specific expression

We tested for expression enrichment of CRS-containing biotypes in specific experiments, e.g., developmental stages and cell lines. For the *k*th biotype *B*_*k*_ and the *l*th modENCODE experiment *E*_*l*_, we define the CRS-ratio *R*_*CRS*_, a non-CRS ratio *R*_¬*CRS*_, and the ratio-difference *R*_*d*_ as

$$ R_{CRS}(B_{k},E_{l}) = \frac{N(B_{k},E_{l},CRS)}{N(B_{k},E,CRS)}, $$

$$ {} R_{\neg CRS}(B_{k},E_{l}) = \frac{N(B_{k},E_{l},\neg CRS)}{N(B_{k},E,\neg CRS)}, $$

2 $$  {\kern10pt}R_{d}(B_{k},E_{l}) = R_{CRS}(B_{k},E_{l}) - R_{\neg CRS}(B_{k},E_{l}),~~~  $$

where *N*(*B*_*k*_,*E*_*l*_,*CRS*) is the number of biotype *B*_*k*_ instances overlapping at least one CRS (minimum overlap of 50% of instance or CRS size) expressed in the currently considered experiment *E*_*l*_, and *N*(*B*_*k*_,*E*,*CRS*) is the respective number expressed in any other experiment *E*. Only instances expressed in at least three experiments and contained in the CMfinder input alignments by at least 50% of their size were considered. For each biotype and each modENCODE experiment, a one-sided Student’s t-test (coding exons, 5’-UTR exons; normally distributed non-CRS ratios) or Wilcoxon-Mann-Whitney test (all other biotypes; non-CRS ratios not normally distributed) was performed to test the significance of deviations of the CRS-ratio from the mean of all non-CRS ratios for that biotype. Depending on the ratio difference being larger or smaller than 0, the alternative hypothesis for the R functions t.test() and wilcox.test() was set to ‘less’ or ‘greater’, respectively. All *p*-values were adjusted for multiple hypothesis testing (Bonferroni). Exon and intron biotypes in this analysis are from the FlyBase annotation (dmel_r6.15, FlyBase release FB2017_02). Promoter annotations were obtained from the EPDnew database [77], enhancer annotations were obtained from the Fly Enhancers database [78], and all non-coding annotations were obtained from FlyBase and, where available, combined with the Rfam annotation (Rfam 12.2). For calculating non-CRS ratios for intergenic regions (FlyBase) we split them into 100 bp long windows and categorized them into bins according to their GC content and sequence identity (in the same way as the CMfinder predictions for the FDR calculation). From each of these bins as many intergenic windows were sampled as there are predictions in that bin.

### Differential expression

To analyze the correlation of RNA structures and differential expression between developmental stages, for each biotype and each modENCODE experiment two expression vectors (with elements of 1 for expression in this experiment, 0 for not being expressed) were generated: one for all biotype instances containing CRSs, and one for instances without CRS. Only instances that are expressed in at least three experiments were considered. Then, for each possible combination of experiment *i* and experiment *j*, the Hamming distance of the respective expression vectors was calculated and normalized by vector length. To describe the impact of RNA secondary structure on the differential expression of developmental stages we define the differential expression score as

3 $$  E_{\text{diff}}(i,j) = D_{H}(CRS) \times |(D_{H}(CRS) - D_{H}(\neg CRS)|, \,  $$

where *D*_*H*_(*CRS*) is the normalized Hamming distance of the two vectors of biotype instances containing at least one CRS and *D*_*H*_(¬*CRS*) is the respective value for biotype instances without CRSs.

## Additional file

Additional file 1 Supplemental Figures. (PDF 5227 kb)

## Abbreviations

CRS: Conserved RNA structure
FDR: False discovery rate
IRES: Internal ribosome entry site
lncRNA: Long non-coding RNA
MAF: Multiple alignment format
miRNA: microRNA
mRNA: Messenger RNA
ncRNA: Non-coding RNA
RBP: RNA binding protein
RNase P: Ribonuclease P
rRNA: Ribosomal RNA
scaRNA: Composite small nucleolar RNA
SECIS: Selenocysteine insertion sequence
sisRNA: Stable intronic sequence RNA
snoRNA: Small nucleolar RNA
snRNA: Small nuclear RNA
tRNA: Transfer RNA
UTR: Untranslated region
