# Supplementary material for: Identification and characterization of novel conserved RNA structures in Drosophila
Source: BMC Genomics. 2018 Dec 11;19:899. doi: 10.1186/s12864-018-5234-4 (PMC6288889; doi:10.1186/s12864-018-5234-4)
Supplement: Supplementary file 1 — Supplemental Figures. (PDF 5227 kb) [file 12864_2018_5234_MOESM1_ESM.pdf]

Identification and Characterization of Novel Conserved RNA Structures in Drosophila

Supplement

Rebecca Kirsch, Stefan E Seemann, Walter L Ruzzo, Stephen M Cohen, Peter F Stadler, Jan Gorodkin

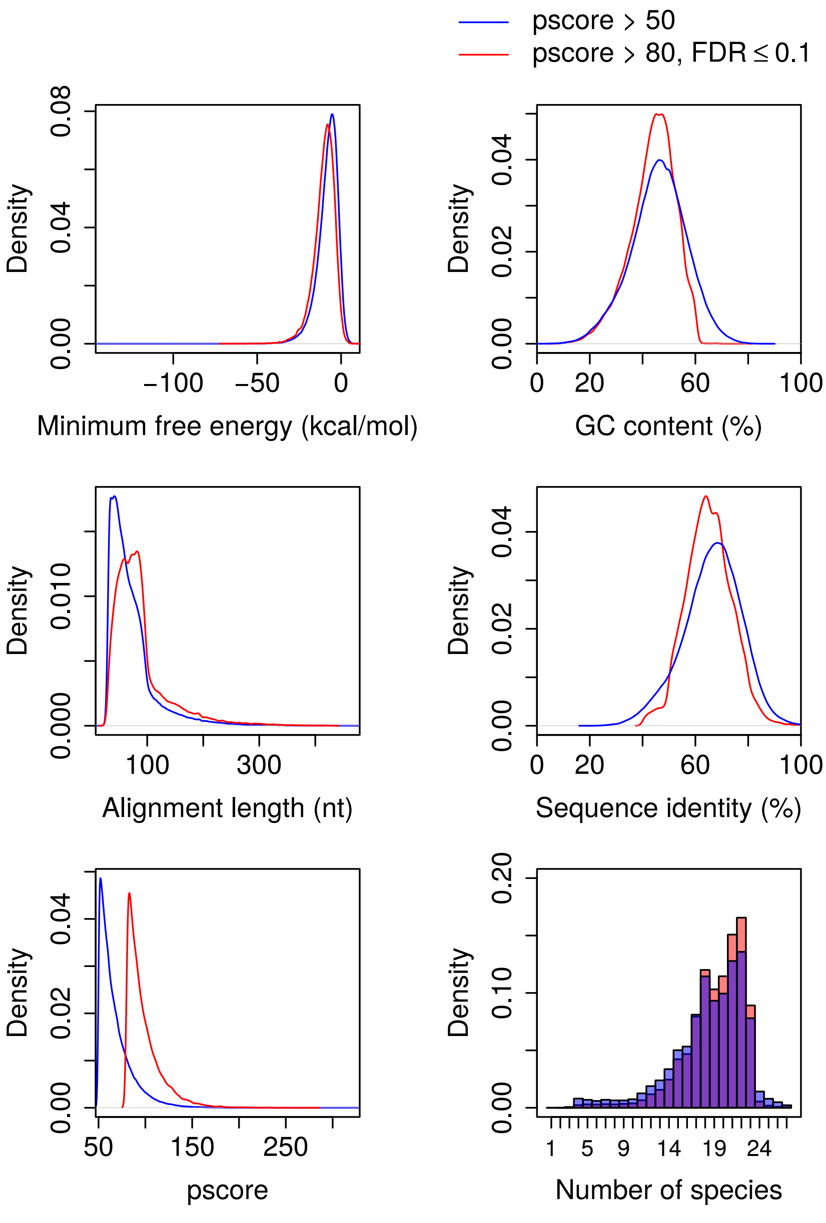

Figure 1: Distribution of selected features of the `CMfinder` hits.

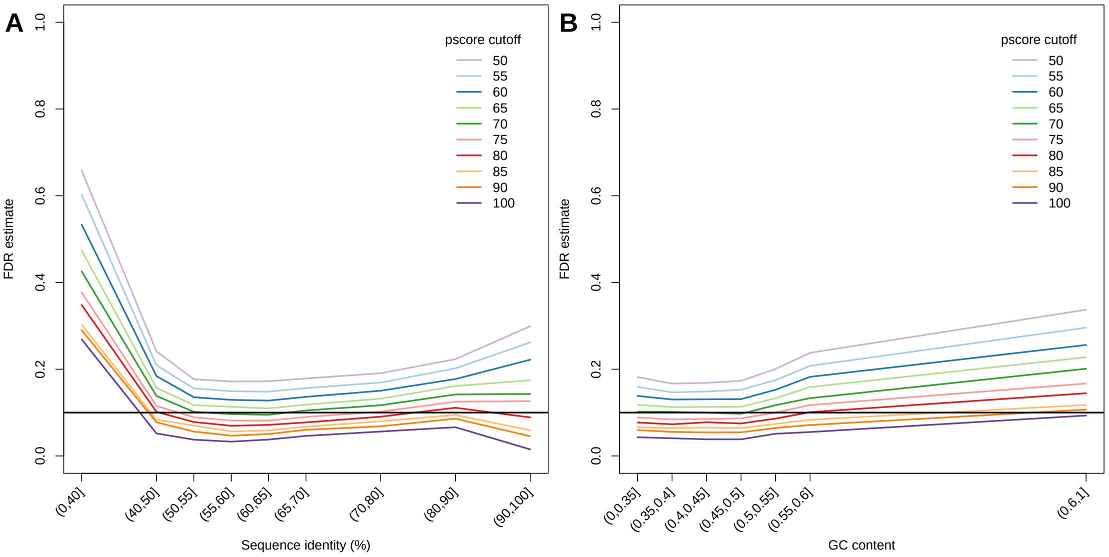

Figure 2: Relationship between mean FDR and sequence identity (A) or GC content (B) of the `CMfinder` predictions, depending on different *pscore* cutoffs.

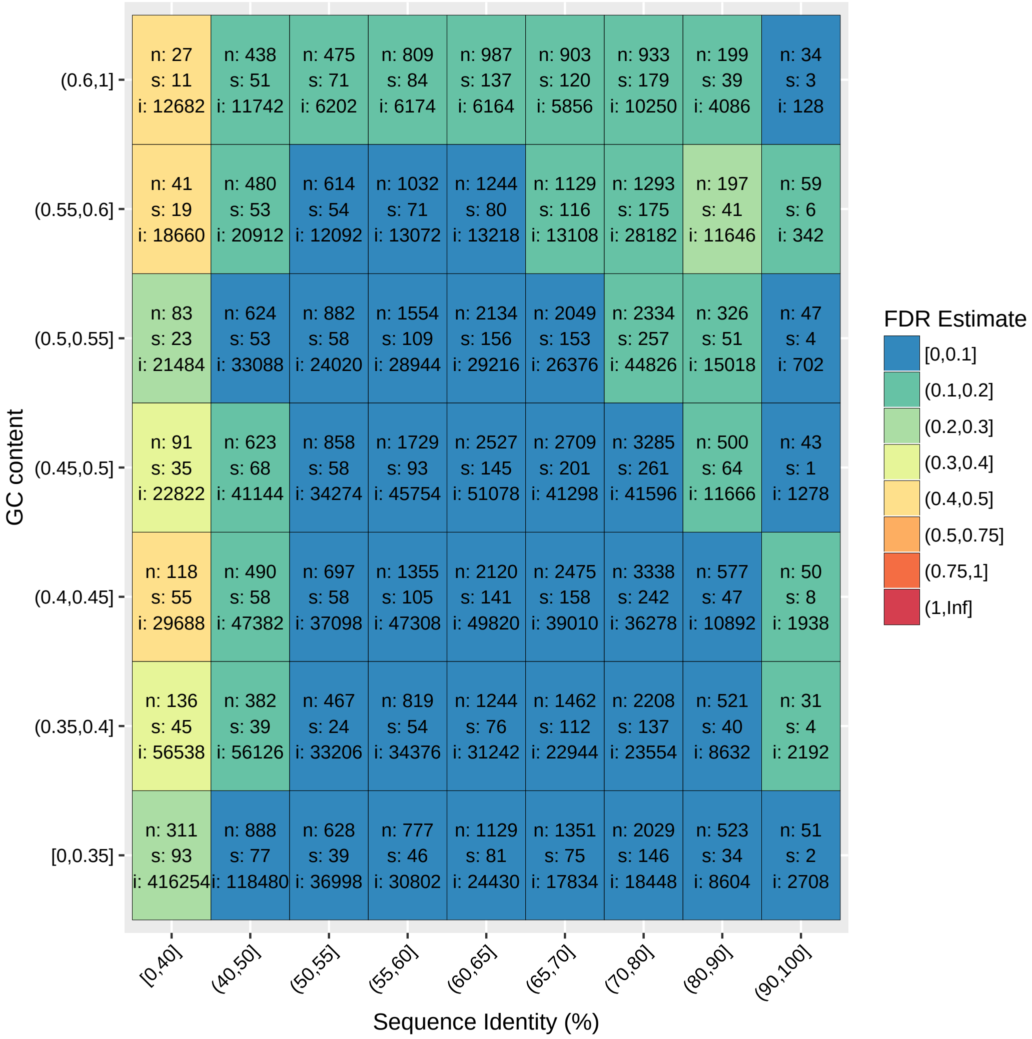

Figure 3: False discovery rates for repeat-filtered `CMfinder` predictions with a *pscore* > 80, depending on their GC content and sequence identity. n: The number of predictions on the native genome in this bin combination. s: The number of predictions on the shuffled genome in this bin combination. i: The number of `CMfinder` input alignment blocks with this GC content and sequence identity, to ensure that all bins have enough potential to harbor predictions and have been chosen meaningfully.

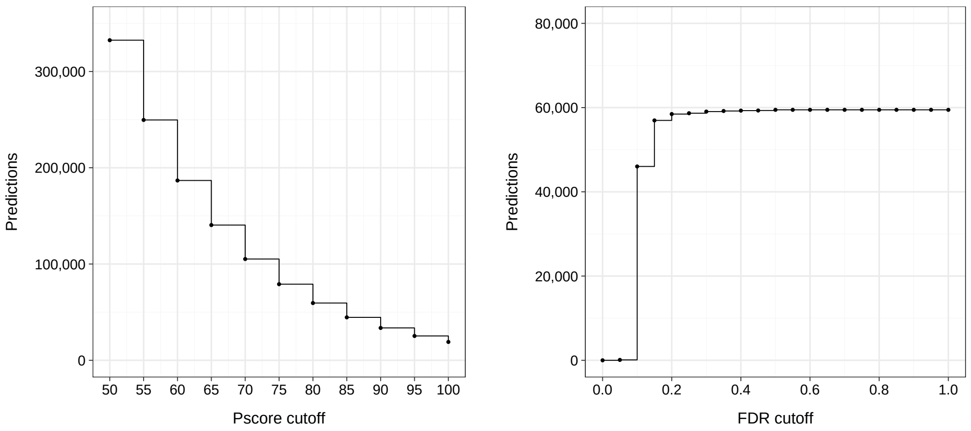

(a) Predictions repeat-filtered.

(b) Predictions repeat-filtered, `pscore` > 80.

Figure 4: Number of `CMfinder` predictions as a function of *pscore* (a) and FDR (b).

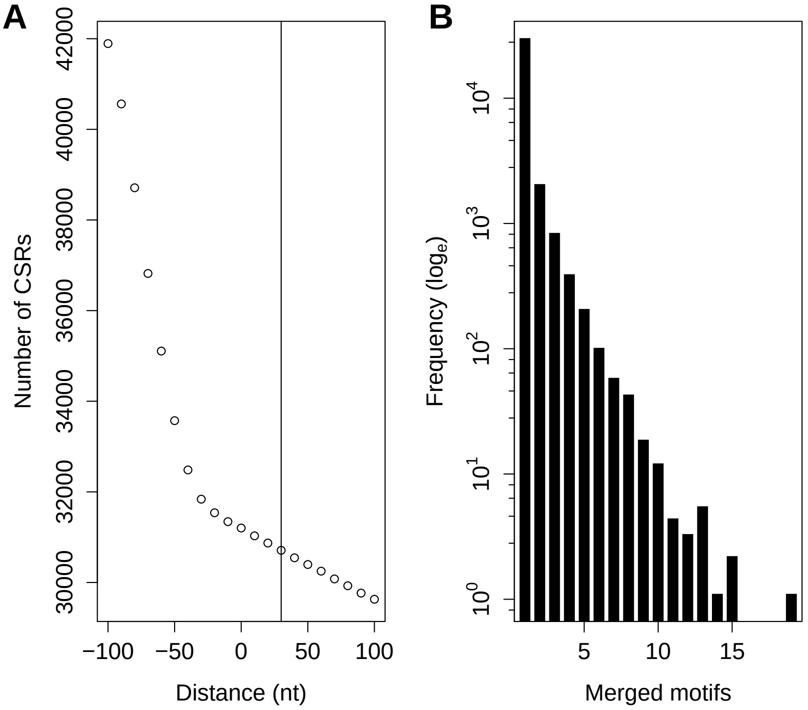

Figure 5: Number of CRSs as a function of the required distance, with negative values corresponding to a minimum overlap (A). When individual predictions are merged at a distance of 30 nt, most of the resulting CRSs consist of one or two formerly individual predictions (B).

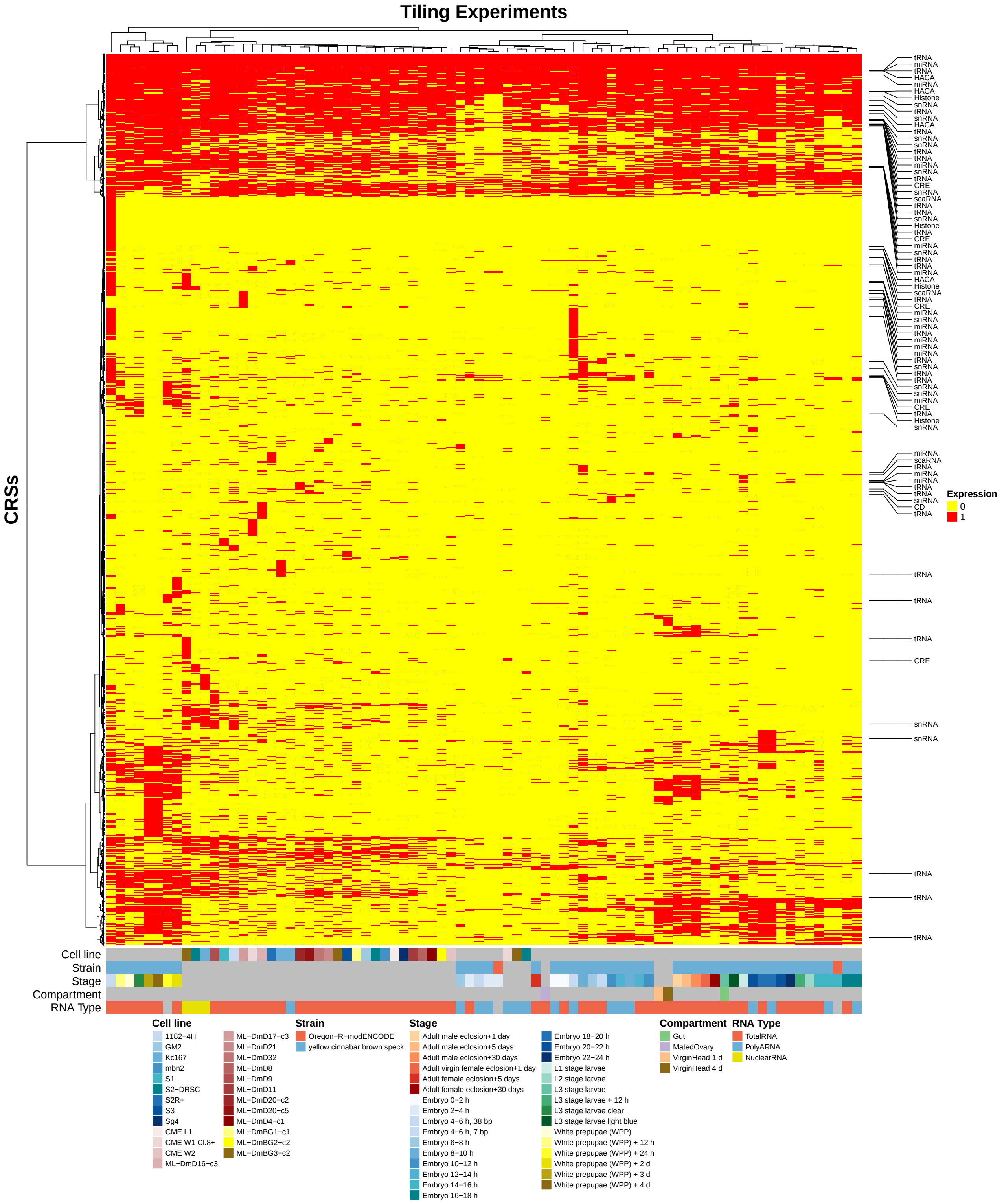

Figure 6: Expression of CRSs according to publicly available modENCODE tiling array expriments. Only CRSs showing at least 50% overlap with at least one transcript region are considered. This version of the figure includes a much more detailed explanation of the included data sets.

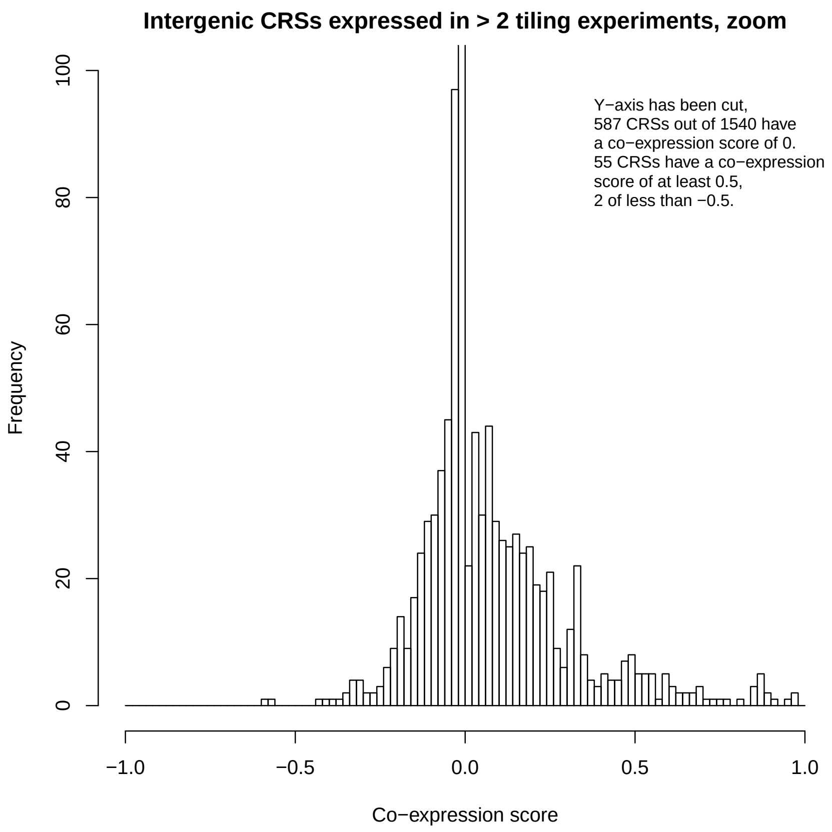

Figure 7: Co-expression of intergenic CRSs with their closest annotated gene element.

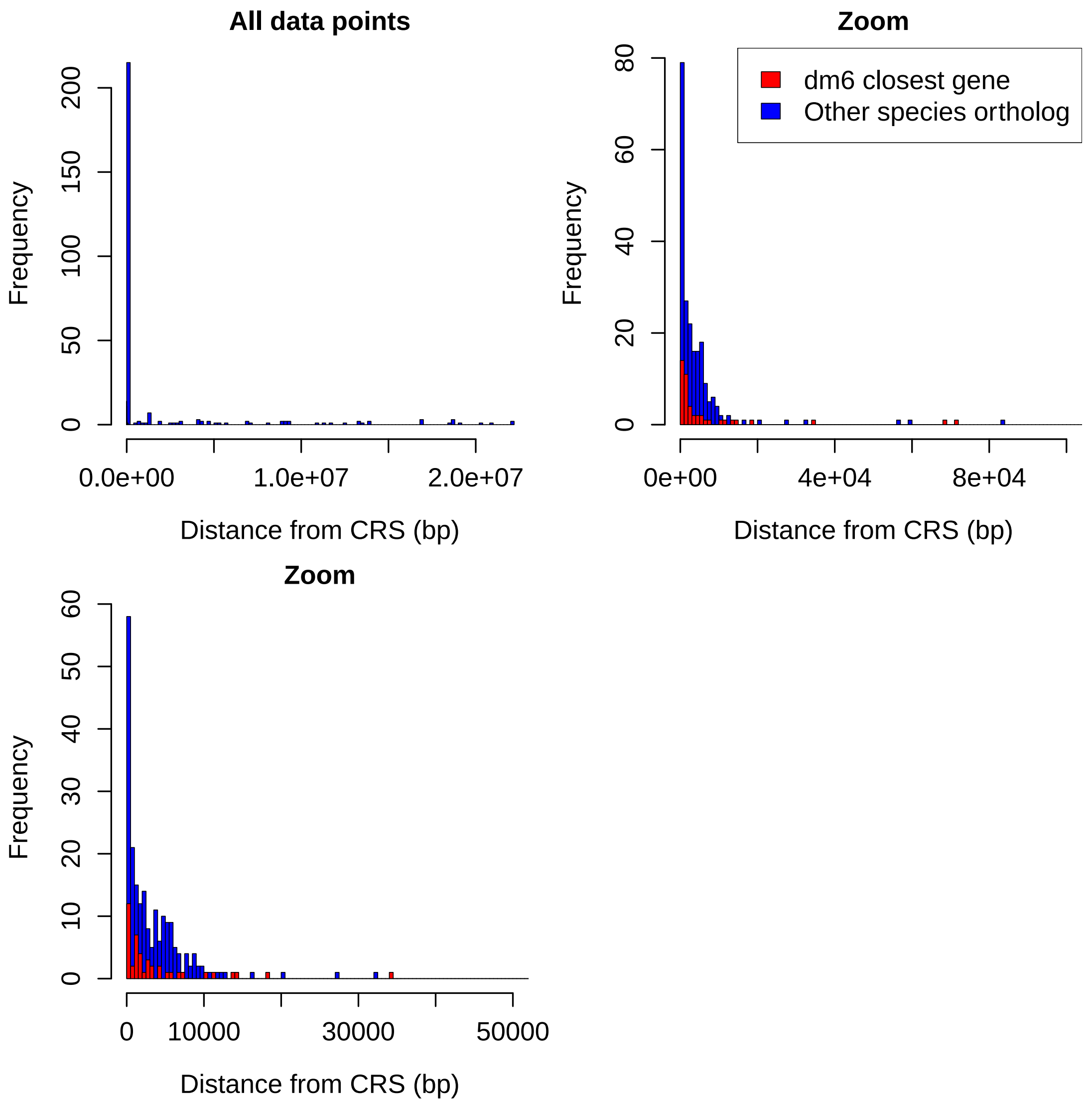

Figure 8: Distances of *D. melanogaster* genes and their orthologs in other drosophilids from intergenic CRSs, for which co-expression was observed. Based on these histograms, a maximal distance of 20 000 bp was chosen to remove very distant orthologs, probably due to major genome rearrangements or mis-annotation, from the synteny analysis investigating orientation of CRS-gene pairs.

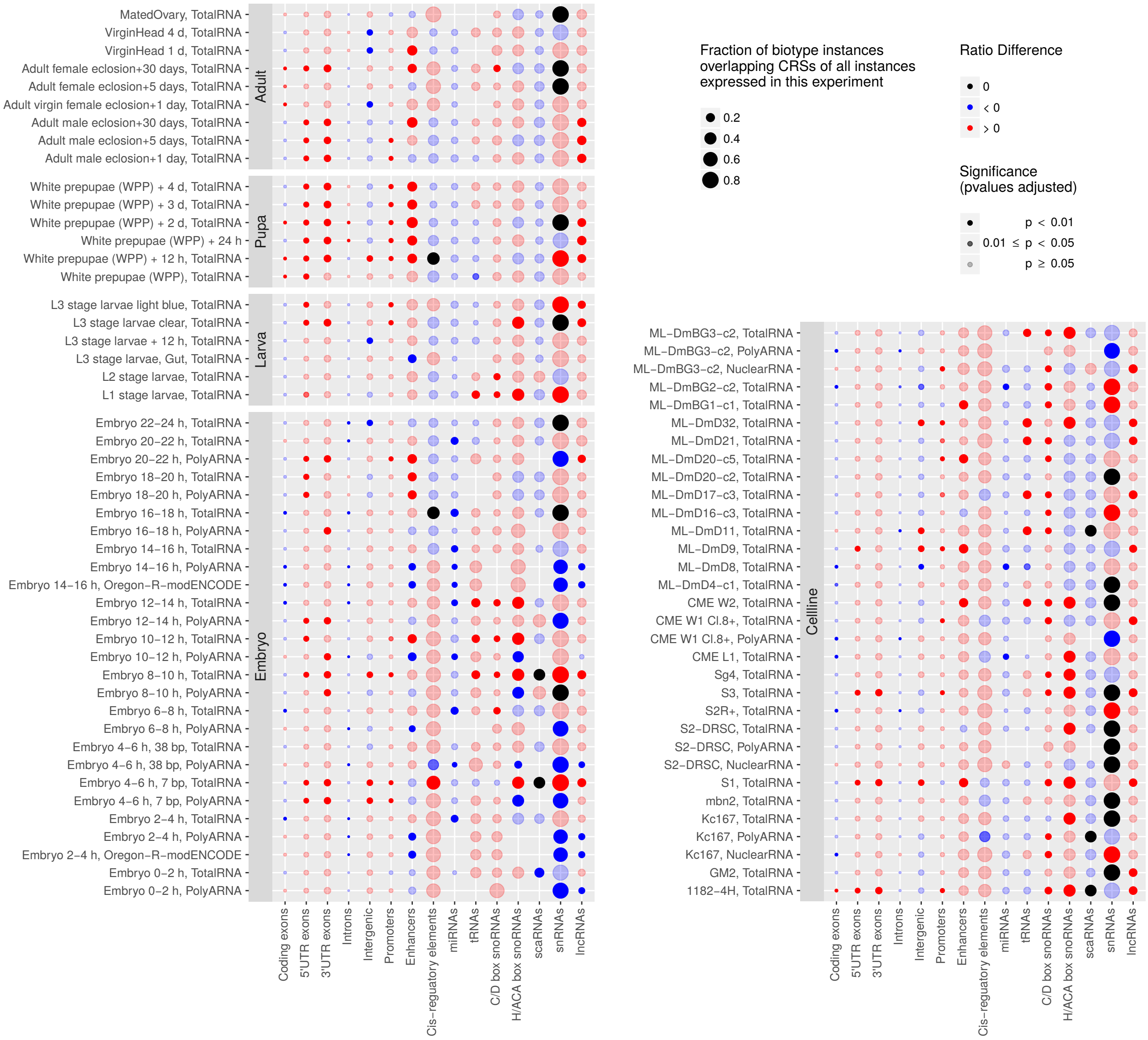

Figure 9: Over- and underrepresentation of biotype instances with CRSs compared to instances without CRSs (‘ratio difference’, color coded) in *Drosophila melanogaster* developmental stages and cell lines. Only instances expressed in at least three tiling array experiments and contained in the `CMfinder` input alignments by at least 50% of the feature size were considered here. Tests of significance (indicated by opacity) assess whether biotype instances with CRSs are expressed more often in a particular stage compared to all other stages than expected by chance. p-values have been adjusted for multiple hypothesis testing (Bonferroni). Unless indicated otherwise, tiling array probes are 38 bp long.
